# Elucidation of the Strongest Predictors of Cardiovascular Events in Patients with Heart Failure

**DOI:** 10.1016/j.ebiom.2018.06.001

**Published:** 2018-06-20

**Authors:** Hiroki Fukuda, Kazuhiro Shindo, Mari Sakamoto, Tomomi Ide, Shintaro Kinugawa, Arata Fukushima, Hiroyuki Tsutsui, Shin Ito, Akira Ishii, Takashi Washio, Masafumi Kitakaze

**Affiliations:** a Department of Clinical Medicine and Development, National Cerebral and Cardiovascular Centre, 5-7-1 Fujishirodai, Suita, Osaka, Japan; b Department of Cell Biology, National Cerebral and Cardiovascular Centre, 5-7-1 Fujishirodai, Suita, Osaka, Japan; c Department of Cardiovascular Medicine, Faculty of Medical Sciences, Kyushu University, 3-1-1 Maidashi, Higashi-ku, Fukuoka, Japan; d Department of Cardiovascular Medicine, Faculty of Medicine, Graduate School of Medicine, Hokkaido University, Japan; e The Institute of Scientific and Industrial Research, Osaka University, 1-1 Yamadaoka, Suita, Osaka, Japan

**Keywords:** Heart failure, Data mining, Cardiovascular events, Combinational factors, Inotropic agents, Diuretics

## Abstract

**Background:**

In previous retrospective studies, we identified the 50 most influential clinical predictors of cardiovascular outcomes in patients with heart failure (HF). The present study aimed to use the novel limitless-arity multiple-testing procedure to filter these 50 clinical factors and thus yield combinations of no more than four factors that could potentially predict the onset of cardiovascular events. A Kaplan–Meier analysis was used to investigate the importance of the combinations.

**Methods:**

In a multi-centre observational trial, we prospectively enrolled 213 patients with HF who were hospitalized because of exacerbation, discharged according to HF treatment guidelines and observed to monitor cardiovascular events. After the observation period, we stratified patients according to whether they experienced cardiovascular events (rehospitalisation or cardiovascular death).

**Findings:**

Among 77,562 combinations of fewer than five clinical parameters, we identified 151 combinations that could potentially explain the occurrence of cardiovascular events. Of these, 145 combinations included the use of inotropic agents, whereas the remaining 6 included the use of diuretics without bradycardia or tachycardia, suggesting that the high probability of cardiovascular events is exclusively determined by these two clinical factors. Importantly, Kaplan–Meier curves demonstrated that the use of inotropes or of diuretics without bradycardia or tachycardia were independent predictors of a markedly worse cardiovascular prognosis.

**Interpretation:**

Patients treated with either inotropic agents or diuretics without bradycardia or tachycardia were at a higher risk of cardiovascular events. The uses of these drugs, regardless of heart rate, are the strongest clinical predictors of cardiovascular events in patients with HF.

**Research in Context**

**Evidence before this study**

Many lines of evidence from the observational or randomised clinical studies have identified the important clinical factors for the prediction of the cardiovascular events by multivariate analyses of observationally collected or randomised controlled data in patients with heart failure (HF), however, there have been no data analyses using many clinical parameters related or unrelated to the pathophysiology of HF patients to seek to the strongest clinical factors by data-mining methods. Here, one of the novel data mining methods of limitless-arity multiple-testing procedure (LANP) could identify the strongest clinical factors to predict the cardiovascular events among all combinations of the clinical factors in HF patients.

We employed 167 HF patients who were admitted between November 2007 and October 2009 and followed to monitor the incidence of cardiovascular events until December 2014 to narrow down 50 important clinical parameters to predict cardiovascular events, and we generated a new cohort of 213 HF patients who received contemporary treatment in the context of a multi-centre trial, and prospectively evaluated the combination that could best predict cardiovascular outcomes between May 2013 and March 2015 and followed these patients until the end of April 2016.

**Added Value of This Study**

Using the LANP method for the patients with HF, we found that the patients treated with either inotropic agents or diuretics without bradycardia or tachycardia were at a higher risk of cardiovascular events, which are novel finding on the top of the conventional knowledge of the current HF treatment strategy.

**Implications of all the Available Evidence**

The cardiologists are usually interested in the symptoms of the patients, results of biomarkers of HF such as plasma BNP levels, laboratory data of echocardiograms and the effectiveness and side-effects of the drugs for HF when they examine the HF patients. On the top of the ordinary knowledge or guidelines of treatment of HF, the present finding cautions that the cardiologists should focus on the present use of inotropic agents or the use of diuretics without either bradycardia or tachycardia as the strongest predictors of an increased risk of cardiovascular events in patients with HF, when cardiologists treat such patients. Such analyses using the big data of HF patients would notify the unexpected parameters to predict the occurrence of the cardiovascular events such as re-hospitalisation.

## 1 Introduction

Globally, cardiovascular disease has placed a significant burden both on individual patients and national economies [1, 2]. Despite the availability of effective medical treatments, heart failure (HF) remains a major cause of increased morbidity and mortality [[3], [4], [5]]. Notably, hospitalisation for a pathophysiologic exacerbation of HF can increase the severity of this condition, thus activating a vicious cycle that leads to cardiovascular death. Therefore, it is very important to identify the strongest clinical predictors of cardiovascular events followed by hospitalisation among patients with HF. Comorbidity (hypertension or renal dysfunction), the presence of anaemia or cardiomegaly, age and sex have been suggested as major determinants of hospitalisation or cardiac death among patients with HF [6]. However, the interactions between these comorbidities are complex, and the strongest clinical influences on the risk of a cardiovascular event remain unclear. In previous studies, several biomarkers, including blood levels of brain natriuretic peptide (BNP) [7], C-reactive protein [8] and albumin [9], have been measured in patients with HF with the aim of determining the severity and probability of cardiovascular events. Additionally, various drugs, such as angiotensin-converting inhibitors [10], diuretics [11] and inotropic agents [12], have been administered to patients with the intent to improve the pathophysiology of HF. Still, it remains difficult to determine the most important clinical predictors of cardiovascular events and to apply this knowledge to patients with HF in a clinical setting.

The existing limitations can be partially attributed to the use of different hypotheses and the lack of comprehensive or systematic investigations among the various studies. Accordingly, it is important to use a comprehensive method to determine the most essential parameters or combinations of parameters predictive of cardiovascular events in a cohort of patients with HF. As the combination of clinical parameters A + B + C may have synergistic effects on cardiovascular events even if A, B or C alone has no effect, the ability of every combination of clinical parameters to predict the occurrence of cardiovascular events should be tested. To overcome the difficulties associated with such testing in patients with HF, we have implemented recent, novel advances in statistical testing that will allow us to analyse all significant combinations of clinical parameters without any limits via the limitless-arity multiple testing procedure (LAMP) [13].

In this study, we evaluated the effects of combinations of clinical parameters on the incidence of cardiovascular events among patients with HF. First, we narrowed down all the combinations to those that could best explain the occurrence of the cardiovascular events. Second, we identified two combinations of clinical parameters, the use of inotropes or the use of diuretics without bradycardia or tachycardia, which correlated with the highest probability of cardiovascular event incidence among patients with HF.

## 2 Methods

### 2.1 Ethics Statement

This study was approved by the National Cerebral and Cardiovascular Centre Research Ethics Committee, which waived the requirement to obtain informed consent from the 167 subjects according to the Japanese Clinical Research Guideline because of the retrospective observational design. Instead, we made a public announcement on both the Internet homepage of our institution and the bulletin boards in our outpatient and inpatient clinics to comply with the Japanese Clinical Research Guideline and a request of the Ethics Committee.

For the analysis, we created a specified database of anonymised data in the Department of HF at our institution and analysed the anonymous data. Additionally, we obtained written informed consent from the 213 subjects included in the prospective observational study after receiving approval from the Research Ethics Committees at the National Cerebral and Cardiovascular Centre, Hokkaido University and Kyushu University.

### 2.2 Protocols for the First and Second Screenings

We filtered the clinical parameters to identify those most important with regard to the incidence of cardiovascular events in patients with HF. Initially, we obtained data of 402 clinical parameters in 151 patients with acute decompensated heart failure (ADHF) and used these data to derive an equation with which to determine the probability of cardiovascular events (hospitalisation or death due to HF) [14]. In this step, we narrowed the list to 251 clinical parameters. Next, after data cleaning, we added 16 patients to the cohort from the previous study to yield a total of 167 patients with ADHF who were admitted between November 2007 and October 2009 and followed to monitor the incidence of cardiovascular events until December 2014. HF diagnoses were confirmed by an expert team of cardiologists using the Framingham criteria. Finally, we selected the 50 most influential candidates from among the 251 parameters identified in previous studies (Table 1) [14, 15].

**Table 1:** The clinical parameters in patients with heart failure, and the differences in the clinical parameters with or without cardiovascular events.

Clinical factors	
Age, (years)	72 (60–79)
Gender, male/female	98/69
NYHA class (II/III/IV) at admission	52/54/61
Heart rate at admission (beats/min)	81 (69–104)
Leg edema	91 (54)
Etiology of HF	
Cardiomyopathy	56 (34)
Hypertensive heart disease	25 (15)
Ischemic heart disease	16 (10)
Valvular heart disease	47 (28)
Comorbidity	
Hypertension	81 (49)
Hyperlipidemia	47 (28)
Chronic Af	67 (40)
Cerebrovascular disease	31 (19)
Obstructive pulmonary disease	10 (6)
CRT	35 (20)
ICD	35 (20)
Pacemaker	14 (8)
Number of family members in the same household	1 (1, 2)
Albumin at admission, (g/dl)	3.7 (3.4–4.0)
CRP at admission, (mg/dl)	0.3 (0.1–0.9)
WBC at admission, (/μl)	6500 (5000–8850)
AST at discharge, (U/l)	25.0 (20.5–21.5)
BUN at discharge, (mg/dl)	21.0 (16–30.8)
Uric acid at discharge, (mg/dl)	7.0 (5.7–8.4)
CRP at discharge, (mg/dl)	0.18 (0.04–0.53)
BNP at discharge, (pg/ml)	191 (102–413)
%FS at admission, (%)	19 (11–29)
LVDs at admission, (mm)	48 (36–57)
%FS at discharge, (%)	20 (13−31)
IVST at discharge, (mm)	9 (8–11)
AR grade (≥II) at discharge	21 (13)
MR grade (≥II) at discharge	48 (29)
TR grade (≥II) at discharge	43 (26)
Oral medications at discharge	
ACE inhibitor	80 (48)
Anti-allergic	12 (7)
Anti-inflammatory drug	5 (3)
Antiplatelet	45 (27)
Antithyroid drug	2 (1)
Beta-blockers	109 (65)
Bronchodilator	7 (4)
Choleretic drug	10 (6)
Digitalis	48 (29)
Diuretics	151 (90)
Inotropic agent	22 (13)
Intestinal disease drug	4 (2)
Lipid-lowering drug	37 (22)
Proton pump inhibitor	60 (36)
Purgative	49 (29)
Sedative-hypnotic (benzodiazepin)	36 (22)
Vitamins	14 (8)

Data are given as the Median (interquartile range) or n (%). ACE inhibitor, angiotensin-converting enzyme inhibitor; ADHF, acute decompensated heart failure; Af, atrial fibrillation; AR, aortic regurgitation; BNP, B-type natriuretic peptide; BUN, Blood urea nitrogen; CRT, cardiac resynchronization therapy; CRP, C-reactive protein; FS, fractional shortening; ICD, Implantable Cardioverter Defibrillator; VST, interventricular septum thickness; LVDs, Left ventricular end-systolic dimension MR, mitral regurgitation; NYHA, New York Heart Association; TR, tricuspid regurgitation.

In the present study, we generated a new cohort of HF patients who received contemporary treatment in the context of a multi-centre trial and prospectively evaluated the combination that could best predict cardiovascular outcomes. For this purpose, we enrolled 213 patients with ADHF who were admitted to three different hospitals in Japan—National Cerebral and Cardiovascular Centre (*n* = 114), Hokkaido University (*n* = 80) and Kyushu University (*n* = 19)—between May 2013 and March 2015 and followed these patients until the end of April 2016. All patients underwent a careful history-taking process, physical examinations, laboratory testing, chest X-rays, electrocardiograms and complete Doppler echocardiographic studies. An expert team of cardiologists in charge of the HF department determined the timing of patient discharge, which was recommended when the patient presented with a stable blood pressure and improved renal function due to an optimal treatment according to international guidelines, as well as none of the following: signs of decompensation such as a New York Heart Association functional class <3, rales and galloping rhythm. Rehospitalisation of HF patients was defined as hospitalisation of an enrolled patient for decompensated HF, and cardiovascular death was defined as death attributed to a worsening of HF. The primary endpoint was a cardiovascular event: either rehospitalisation or death due to a worsening of HF, whichever occurred first. Among the 50 clinical parameters, we determined the left ventricular dimensions at diastole and systole from the calculated of percent fractional shortening. As we included additional parameters related to the etiology of HF, such as cardiomyopathy (Table 2), the LAMP analysis actually included 54 clinical parameters at the time of hospitalisation or discharge in HF patients.

**Table 2:** The clinical parameters in patients with heart failure, and the differences in the clinical parameters with or without cardiovascular events.

Clinical factors	Without (n = 114)	With (n = 99)
Age, (years)	72 (60–79)	70 (60–79)
Gender, male/female	71/43	64/35
NYHA class (II/III/IV) at admission	34/55/25	13/53/33
Heart rate at admission (beats/min)	86 (69–102)	75 (69–87)
Leg edema	65 (57)	71 (62)
Etiology of HF		
Cardiomyopathy	34 (30)	42 (37)
Hypertensive heart disease	23 (20)	6 (5)
Ischemic heart disease	12 (11)	14 (12)
Valvular heart disease	23 (20)	24 (21)
Others	22 (19)	13 (11)
Comorbidity		
Hypertension	64 (56)	44 (39)
Hyperlipidemia	40 (35)	33 (29)
Chronic Af	50 (44)	54 (47)
Cerebrovascular disease	7 (6)	7 (6)
Obstructive pulmonary disease	5 (4)	1 (1)
CRT	8 (7)	16 (14)
ICD	11 (10)	20 (18)
Pacemaker	18 (16)	13 (11)
Number of family members in the same household	1 (1, 2)	1 (1)
Albumin at admission, (g/dl)	3.8 (3.5–4.1)	3.8 (3.5–4.1)
CRP at admission, (mg/dl)	0.4 (0.1–1.2)	0.4 (0.15–1.05)
WBC at admission, (/μl)	5300 (4100–6369)	5100 (4200–6700)
AST at discharge, (U/l)	20 (18–28)	25 (20−32)
BUN at discharge, (mg/dl)	22 (18–28)	27 (20.5–44)
Uric acid at discharge, (mg/dl)	6.4 (5.3–7.6)	6.8 (5.3–8.1)
CRP at discharge, (mg/dl)	0.1 (0.1–0.4)	0.2 (0.1–0.7)
BNP at discharge, (pg/ml)	196 (117–407)	294 (165–534)
%FS at admission	18.8 (10.1–29.1)	17.2 (9.7–32.1)
LVDd at admission	58 (49–65)	58 (48–67)
LVDs at admission, (mm)	47 (34–57)	47 (32–58)
%FS at discharge, (%)	21.8 (10.5–31.5)	19 (10−32)
LVDd at discharge	57 (49–63)	59 (48–68)
LVDs at discharge	45 (33–54)	47 (32–60)
IVST at discharge, (mm)	10 (8–11)	10 (8–11)
AR grade (≥II) at discharge	13 (11)	13 (11)
MR grade (≥II) at discharge	45 (39)	48 (42)
TR grade (≥II) at discharge	24 (21)	35 (31)
Oral medications at discharge		
ACE inhibitor	66 (58)	45 (39)
Anti-allergic	3 (3)	5 (4)
Anti-inflammatory drug	25 (22)	23 (20)
Antiplatelet	17 (15)	10 (9)
Antithyroid drug	1 (1)	2 (2)
Beta-blockers	88 (77)	73 (64)
Broncodilator	0 (0)	2 (2)
Choleretic drug	4 (4)	7 (6)
Digitalis	16 (14)	26 (23)
Diuretics	89 (78)	92 (81)
Inotropic agent	4 (4)	32 (28)
Intestinal disease drug	5 (4)	14 (12)
Lipid-lowering drug	44 (39)	35 (31)
Proton pump inhibitor	62 (54)	57 (50)
Purgative	28 (25)	35 (31)
Sedative-hypnotic (benzodiazepin)	6 (5)	6 (5)
Vitamins	3	4 (4)

Data are given as the Median (interquartile range) or n (%). ACE inhibitor, angiotensin-converting enzyme inhibitor; ADHF, acute decompensated heart failure; Af, atrial fibrillation; AR, aortic regurgitation; BNP, B-type natriuretic peptide; BUN, Blood urea nitrogen; CRT, cardiac resynchronization therapy; CRP, C-reactive protein; FS, fractional shortening; ICD, Implantable Cardioverter Defibrillator; VST, interventricular septum thickness; LVDs, Left ventricular end-systolic dimension MR, mitral regurgitation; NYHA, New York Heart Association; TR, tricuspid regurgitation.

### 2.3 Analytic Procedures for the Third Screening

All data related to the events prior to discharge were evaluated in our investigation of the known or unknown factors that contribute to cardiovascular events and are listed in Table 1. We used the novel LAMP to our data-mining initiative to identify both single factors and combinations of factors that would significantly affect the occurrence of cardiovascular events [13]. In our analysis, a patient with HF was represented by both individual clinical factors and the class labels of groups with or without cardiovascular events, and the set of the patients was used to form a data table in which each row represented a patient. This data table D comprises N rows, each of which consists of M factors and a positive or negative class label of an object. Accordingly, LAMP uses Fisher's exact test to draw conclusions from a complete set of statistically significant hypotheses regarding a class label. Here, the hypothesis is based on a combination of a class label and a condition defined as a subset of the M factors in D. As the condition of the uncovered significant hypothesis may include any number of factors from 1 to M, the term ‘limitless-arity’ has been used to describe this method. Accordingly, LAMP applies a highly efficient search algorithm to quickly and completely derive significant hypotheses from 2^M^ candidates.

If k is the number of all hypotheses for which the conditions exceed or remain equal to σ objects in D (σ < N), the relationship between k and σ, k = k_D_(σ) depends on D but is always anti-monotonic because fewer hypothesis conditions remain true at a higher frequency in D. Although the formula of k_D_(σ) is not analytically determined, LAMP includes a mining algorithm used to efficiently derive all k hypothesis conditions under a given σ. The Bonferroni correction, which sets a boundary for the family-wise error rate of the false negative in the multiple tests at <1 significance level α by correcting the level to α/k_D_(σ), can be used as a standard multiple testing procedure for the k hypotheses. Note that this level is monotonic to σ, as k_D_(σ) is anti-monotonic. If we use a very small set value of σ for a complete search of the significant hypotheses, α/k_D_(σ) is extremely small because k_D_(σ) approaches 2^M^. In this scenario, almost no hypotheses will be accepted as significant. Conversely, if the set value of σ and, consequently, α/k_D_(σ) is too large, k_D_(σ) will be very small and some significant hypothesis conditions will be missed. To overcome this limitation, LAMP uses the fact that any hypothesis with a frequency less than σ will not have a *p* value less than the following level.

$$f\left(\sigma \right)=\left(\begin{array}{c} n_{p}\\ \sigma \end{array}\right)/\left(\begin{array}{c} N\\ \sigma \end{array}\right)$$

Here, n_p_ is the number of the objects with positive class labels in D (n_p_ < N). Accordingly, any hypothesis with a frequency less than σ will not be accepted if f(σ) > α/k_D_(σ). Because f(σ) is anti-monotonic for σ and α/k_D_(σ) is monotonic, LAMP selects σ^⁎^ to balance f(σ^⁎^) and α/k_D_(σ^⁎^). The selected value of σ^⁎^ yields the smallest number of candidate hypotheses without applying the tests or missing any significant hypotheses.

For practical reasons, we were interested in a hypothesis that would hold true for at least 19 patients. As all hypotheses involving more than four factors failed to meet this criterion, we limited our LAMP-based search of the hypotheses to a maximum of four factors. This limitation further reduced the number k_D_(σ^⁎^) of the candidate hypotheses and increased the level α/k_D_(σ^⁎^) in LAMP. After we obtained all significant hypotheses regarding single clinical factors or combinations of factors, we excluded each hypothesis for which the condition was a superset of the conditions from other simpler hypotheses, as the significance of the former would be trivial in comparison with the significance of the latter. Once we had narrowed down all single or combination clinical parameters to single or combinational clinical factors, we used a Kaplan–Meier analysis to test whether these clinical factors could predict cardiovascular events among the enrolled patients.

## 3 Results

Table 1 lists the patients' clinical characteristics, whereas Table 2 stratifies the characteristics of patients who did and did not experience cardiovascular events. We next performed a LAMP analysis that maintained the family-wise error rate below the required significance level by calibrating the Bonferroni factor to examine the significant combinations of these 54 clinical parameters and thus characterised the cardiovascular outcomes. In our analysis of 77,562 combinations with no >4 clinical parameters, we identified 151 combinations involving 54 parameters that predicted the occurrence of cardiovascular events (Table 3). Among these 151 combinations, 145 included the use of inotropic agents as a factor, which was also found to significantly correlate with the occurrence of cardiovascular events as a single factor (Rank 1 in Table 3). Therefore, we pooled all ranks that included the use of inotropic agents (Category 1 in Table 4). Of the remaining six combinations (Category 2 in Table 4), all included the use of diuretics without either bradycardia or tachycardia as a factor. We defined either tachycardia and bradycardia as heart rate >100/min or <50/min. As none of the combinations excluded both of these factors (Table 4), this suggests that the use of inotropic agents or the use of diuretics without either bradycardia or tachycardia may be the most essential clinical factors predictive of the likelihood of cardiovascular events in patients with HF.

**Table 3:** The combinations of clinical parameters to predict the occurrence of the cardiovascular events.

Rank	The combination of clinical parameters				Adjusted *p*-value
1	The use of inotropic agents				0.00071
2	The use of diuretics	The use of inotropic agents			0.00071
3	The use of diuretics	The use of inotropic agents	The abnormal value of bnp (18.4 pg/ml <) at discharge		0.00071
4	The use of inotropic agents	The abnormal value of bnp (18.4 pg/ml <) at discharge			0.00071
5	The use of diuretics	In nyha class iii or ivat admission	Without either tachycardia (100 bpm <) or bradycardia(<50 bpm)		0.00237
6	The use of diuretics	In nyha class iii or ivat admission	Without either tachycardia (100 bpm <) or bradycardia(<50 bpm)	The abnormal value of bnp (18.4 pg/ml <) at discharge	0.00237
7	The use of diuretics	In nyha class iii or ivat admission	Without either tachycardia (100 bpm <) or bradycardia(<50 bpm)	Living with family members in the same household	0.00262
8	The use of inotropic agents	In nyha class iii or ivat admission			0.00383
9	The use of inotropic agents	The abnormal value of bnp (18.4 pg/ml <) at discharge	In nyha class iii or ivat admission		0.00383
10	The use of diuretics	The use of inotropic agents	The abnormal value of bnp (18.4 pg/ml <) at discharge	In nyha class iii or ivat admission	0.00383
11	The use of diuretics	The use of inotropic agents	In nyha class iii or ivat admission		0.00383
12	The use of inotropic agents	The abnormal value of lvds (34 mm <) at discharge			0.00383
13	The use of inotropic agents	The abnormal value of bnp (18.4 pg/ml <) at discharge	The abnormal value of lvds (34 mm <) at admission		0.00383
14	The use of diuretics	The use of inotropic agents	The abnormal value of bnp (18.4 pg/ml <) at discharge	The abnormal value of lvds (34 mm <) at admission	0.00383
15	The use of diuretics	The use of inotropic agents	The abnormal value of lvds (34 mm <) at admission		0.00383
16	The use of inotropic agents	The abnormal value of lvds (34 mm <) at discharge			0.00383
17	The use of inotropic agents	The abnormal value of bnp (18.4 pg/ml <) at discharge	The abnormal value of lvds (34 mm <) at discharge		0.00383
18	The use of diuretics	The use of inotropic agents	The abnormal value of bnp (18.4 pg/ml <) at discharge	The abnormal value of lvds (34 mm <) at discharge	0.00383
19	The use of diuretics	The use of inotropic agents	The abnormal value of lvds (34 mm <) at discharge		0.00383
20	The use of inotropic agents	The abnormal value of %fs (<30%) at discharge			0.00383
21	The use of inotropic agents	The abnormal value of %fs (<30%) at discharge	The abnormal value of bnp (18.4 pg/ml <) at discharge		0.00383
22	The use of diuretics	The use of inotropic agents	The abnormal value of %fs (<30%) at discharge	The abnormal value of bnp (18.4 pg/ml <) at discharge	0.00383
23	The use of diuretics	The use of inotropic agents	The abnormal value of %fs (<30%) at discharge		0.00383
24	The abnormal value of %FS (<30%) at admission	The use of inotropic agents			0.00871
25	The abnormal value of %FS (<30%) at admission	The use of inotropic agents	The abnormal value of lvds (34 mm <) at admission		0.00871
26	The abnormal value of %FS (<30%) at admission	The use of inotropic agents	The abnormal value of BNP (18.4 pg/ml <) at discharge	The abnormal value of lvds (34 mm <) at admission	0.00871
27	The abnormal value of %FS (<30%) at admission	The use of inotropic agents	The use of diuretics	The abnormal value of lvds (34 mm <) at admission	0.00871
28	The abnormal value of %FS (<30%) at admission	The use of inotropic agents	The abnormal value of BNP (18.4 pg/ml <) at discharge		0.00871
29	The abnormal value of %FS (<30%) at admission	The use of inotropic agents	The use of diuretics	The abnormal value of BNP (18.4 pg/ml <) at discharge	0.00871
30	The abnormal value of %FS (<30%) at admission	The use of inotropic agents	The use of diuretics		0.00871
31	The use of inotropic agents	The abnormal value of lvds (34 mm <) at discharge	The abnormal value of lvds (34 mm <) at discharge		0.00871
32	The use of inotropic agents	The abnormal value of lvds (34 mm <) at discharge	The abnormal value of bnp (18.4 pg/ml <) at discharge	The abnormal value of lvds (34 mm <) at discharge	0.00871
33	The use of diuretics	The use of inotropic agents	The abnormal value of lvds (34 mm <) at admission	The abnormal value of lvds (34 mm <) at discharge	0.00871
34	The use of inotropic agents	The abnormal value of %fs (<30%) at discharge	The abnormal value of lvds (34 mm <) at admission		0.00871
35	The use of inotropic agents	The abnormal value of %fs (<30%) at discharge	The abnormal value of bnp (18.4 pg/ml <) at discharge	The abnormal value of lvds (34 mm <) at admission	0.00871
36	The use of diuretics	The use of inotropic agents	The abnormal value of %fs (<30%) at discharge	The abnormal value of lvds (34 mm <) at admission	0.00871
37	The abnormal value of %FS (<30%) at admission	The use of inotropic agents	The abnormal value of %FS (<30%) at discharge		0.00871
38	The abnormal value of %FS (<30%) at admission	The use of inotropic agents	The abnormal value of %FS (<30%) at discharge	The abnormal value of lvds (34 mm <) at admission	0.00871
39	The abnormal value of %FS (<30%) at admission	The use of inotropic agents	The abnormal value of %FS (<30%) at discharge	The abnormal value of BNP (18.4 pg/ml <) at discharge	0.00871
40	The abnormal value of %FS (<30%) at admission	The use of inotropic agents	The abnormal value of %FS (<30%) at discharge	The use of diuretics	0.00871
41	The use of inotropic agents	The abnormal value of %fs (<30%) at discharge	The abnormal value of lvds (34 mm <) at discharge		0.00871
42	The use of inotropic agents	The abnormal value of %fs (<30%) at discharge	The abnormal value of bnp (18.4 pg/ml <) at discharge	The abnormal value of lvds (34 mm <) at discharge	0.00871
43	The use of diuretics	The use of inotropic agents	The abnormal value of %fs (<30%) at discharge	The abnormal value of lvds (34 mm <) at discharge	0.00871
44	The use of inotropic agents	The abnormal value of lvdd (52 mm <) at discharge			0.00871
45	The use of inotropic agents	The abnormal value of lvdd (52 mm <) at discharge	The abnormal value of bnp (18.4 pg/ml <) at discharge		0.00871
46	The use of diuretics	The use of inotropic agents	The abnormal value of lvdd (52 mm <) at discharge	The abnormal value of bnp (18.4 pg/ml <) at discharge	0.00871
47	The use of diuretics	The use of inotropic agents	The abnormal value of lvdd (52 mm <) at discharge		0.00871
48	The use of diuretics	Without either tachycardia (100 bpm <) or bradycardia(<50 bpm)			0.01388
49	The use of inotropic agents	With leg edema			0.01857
50	The use of inotropic agents	With leg edema	The abnormal value of bnp (18.4 pg/ml <) at discharge		0.01857
51	The use of diuretics	The use of inotropic agents	With leg edema	The abnormal value of bnp (18.4 pg/ml <) at discharge	0.01857
52	The use of diuretics	The use of inotropic agents	With leg edema		0.01857
53	The use of diuretics	The abnormal value of bnp (18.4 pg/ml <) at discharge	Without either tachycardia (100 bpm <) or bradycardia(<50 bpm)		0.01873
54	The use of inotropic agents	Without either tachycardia (100 bpm <) or bradycardia(<50 bpm)			0.0196
55	The use of inotropic agents	Without either tachycardia (100 bpm <) or bradycardia(<50 bpm)	The abnormal value of bnp (18.4 pg/ml <) at discharge		0.0196
56	The use of diuretics	The use of inotropic agents	Without either tachycardia (100 bpm <) or bradycardia(<50 bpm)	The abnormal value of bnp (18.4 pg/ml <) at discharge	0.0196
57	The use of diuretics	The use of inotropic agents	Without either tachycardia (100 bpm <) or bradycardia(<50 bpm)		0.0196
58	The use of inotropic agents	In nyha class iii or ivat admission	The abnormal value of lvds (34 mm <) at admission		0.0196
59	The use of inotropic agents	In nyha class iii or ivat admission	The abnormal value of bnp (18.4 pg/ml <) at discharge	The abnormal value of lvds (34 mm <) at admission	0.0196
60	The use of diuretics	The use of inotropic agents	In nyha class iii or ivat admission	The abnormal value of lvds (34 mm <) at admission	0.0196
61	The use of inotropic agents	In nyha class iii or ivat admission	The abnormal value of lvds (34 mm <) at discharge		0.0196
62	The use of inotropic agents	In nyha class iii or ivat admission	The abnormal value of bnp (18.4 pg/ml <) at discharge	The abnormal value of lvds (34 mm <) at discharge	0.0196
63	The use of diuretics	The use of inotropic agents	In nyha class iii or ivat admission	The abnormal value of lvds (34 mm <) at discharge	0.0196
64	The abnormal value of %FS (<30%) at admission	The use of inotropic agents	The abnormal value of lvds (34 mm <) at discharge		0.0196
65	The abnormal value of %FS (<30%) at admission	The use of inotropic agents	The abnormal value of lvds (34 mm <) at admission	The abnormal value of lvds (34 mm <) at discharge	0.0196
66	The abnormal value of %FS (<30%) at admission	The use of inotropic agents	The abnormal value of BNP (18.4 pg/ml <) at discharge	The abnormal value of lvds (34 mm <) at discharge	0.0196
67	The abnormal value of %FS (<30%) at admission	The use of inotropic agents	The use of diuretics	The abnormal value of lvds (34 mm <) at discharge	0.0196
68	The use of inotropic agents	The abnormal value of %fs (<30%) at discharge	In nyha class iii or ivat admission		0.0196
69	The use of inotropic agents	The abnormal value of %fs (<30%) at discharge	The abnormal value of bnp (18.4 pg/ml <) at discharge	In nyha class iii or ivat admission	0.0196
70	The use of diuretics	The use of inotropic agents	The abnormal value of %fs (<30%) at discharge	In nyha class iii or ivat admission	0.0196
71	The use of inotropic agents	The abnormal value of %fs (<30%) at discharge	The abnormal value of lvds (34 mm <) at admission	The abnormal value of lvds (34 mm <) at discharge	0.0196
72	The abnormal value of %FS (<30%) at admission	The use of inotropic agents	The abnormal value of %FS (<30%) at discharge	The abnormal value of lvds (34 mm <) at discharge	0.0196
73	The use of inotropic agents	The abnormal value of lvdd (52 mm <) at discharge	The abnormal value of lvds (34 mm <) at admission		0.0196
74	The use of inotropic agents	The abnormal value of lvdd (52 mm <) at discharge	The abnormal value of bnp (18.4 pg/ml <) at discharge	The abnormal value of lvds (34 mm <) at admission	0.0196
75	The use of diuretics	The use of inotropic agents	The abnormal value of lvdd (52 mm <) at discharge	The abnormal value of lvds (34 mm <) at admission	0.0196
76	The use of inotropic agents	The abnormal value of lvdd (52 mm <) at discharge	The abnormal value of lvds (34 mm <) at discharge		0.0196
77	The use of inotropic agents	The abnormal value of lvds (34 mm <) at discharge	The abnormal value of lvdd (52 mm <) at discharge	The abnormal value of lvds (34 mm <) at discharge	0.0196
78	The use of inotropic agents	The abnormal value of lvdd (52 mm <) at discharge	The abnormal value of bnp (18.4 pg/ml <) at discharge	The abnormal value of lvds (34 mm <) at discharge	0.0196
79	The use of diuretics	The use of inotropic agents	The abnormal value of lvdd (52 mm <) at discharge	The abnormal value of lvds (34 mm <) at discharge	0.0196
80	The use of inotropic agents	The abnormal value of lvdd (52 mm <) at discharge			0.0196
81	The use of inotropic agents	The abnormal value of lvdd (52 mm <) at discharge	The abnormal value of lvdd (52 mm <) at discharge		0.0196
82	The use of inotropic agents	The abnormal value of lvdd (52 mm <) at discharge	The abnormal value of lvdd (52 mm <) at discharge	The abnormal value of lvds (34 mm <) at discharge	0.0196
83	The use of inotropic agents	The abnormal value of lvdd (52 mm <) at discharge	The abnormal value of lvdd (52 mm <) at discharge	The abnormal value of lvds (34 mm <) at admission	0.0196
84	The use of inotropic agents	The abnormal value of lvdd (52 mm <) at discharge	The abnormal value of lvdd (52 mm <) at discharge	The abnormal value of bnp (18.4 pg/ml <) at discharge	0.0196
85	The use of diuretics	The use of inotropic agents	The abnormal value of lvdd (52 mm <) at discharge	The abnormal value of lvdd (52 mm <) at discharge	0.0196
86	The use of inotropic agents	The abnormal value of lvdd (52 mm <) at discharge	The abnormal value of lvds (34 mm <) at discharge		0.0196
87	The use of inotropic agents	The abnormal value of lvds (34 mm <) at discharge	The abnormal value of lvdd (52 mm <) at discharge	The abnormal value of lvds (34 mm <) at discharge	0.0196
88	The use of inotropic agents	The abnormal value of bnp (18.4 pg/ml <) at discharge	The abnormal value of lvdd (52 mm <) at discharge	The abnormal value of lvds (34 mm <) at discharge	0.0196
89	The use of diuretics	The use of inotropic agents	The abnormal value of lvdd (52 mm <) at discharge	The abnormal value of lvds (34 mm <) at discharge	0.0196
90	The use of inotropic agents	The abnormal value of lvdd (52 mm <) at discharge	The abnormal value of lvds (34 mm <) at admission		0.0196
91	The use of inotropic agents	The abnormal value of bnp (18.4 pg/ml <) at discharge	The abnormal value of lvdd (52 mm <) at discharge	The abnormal value of lvds (34 mm <) at admission	0.0196
92	The use of diuretics	The use of inotropic agents	The abnormal value of lvdd (52 mm <) at discharge	The abnormal value of lvds (34 mm <) at admission	0.0196
93	The use of inotropic agents	The abnormal value of lvdd (52 mm <) at discharge	The abnormal value of bnp (18.4 pg/ml <) at discharge		0.0196
94	The use of diuretics	The use of inotropic agents	The abnormal value of lvdd (52 mm <) at discharge	The abnormal value of bnp (18.4 pg/ml <) at discharge	0.0196
95	The use of diuretics	The use of inotropic agents	The abnormal value of lvdd (52 mm <) at discharge		0.0196
96	The use of inotropic agents	With leg edema	In nyha class iii or ivat admission		0.04253
97	The use of inotropic agents	With leg edema	The abnormal value of bnp (18.4 pg/ml <) at discharge	In nyha class iii or ivat admission	0.04253
98	The use of diuretics	The use of inotropic agents	With leg edema	In nyha class iii or ivat admission	0.04253
99	The use of inotropic agents	Living with family members in the same household			0.04356
100	The use of inotropic agents	The abnormal value of bnp (18.4 pg/ml <) at discharge	Living with family members in the same household		0.04356
101	The use of diuretics	The use of inotropic agents	The abnormal value of bnp (18.4 pg/ml <) at discharge	Living with family members in the same household	0.04356
102	The use of diuretics	The use of inotropic agents	Living with family members in the same household		0.04356
103	The use of inotropic agents	The use of beta-blockers			0.04356
104	The use of inotropic agents	The abnormal value of bnp (18.4 pg/ml <) at discharge	The use of beta-blockers		0.04356
105	The use of diuretics	The use of inotropic agents	The abnormal value of bnp (18.4 pg/ml <) at discharge	The use of beta-blockers	0.04356
106	The use of diuretics	The use of inotropic agents	The use of beta-blockers		0.04356
107	The use of inotropic agents	Without either tachycardia (100 bpm <) or bradycardia(<50 bpm)	The abnormal value of lvds (34 mm <) at admission		0.04356
108	The use of inotropic agents	Without either tachycardia (100 bpm <) or bradycardia(<50 bpm)	The abnormal value of bnp (18.4 pg/ml <) at discharge	The abnormal value of lvds (34 mm <) at admission	0.04356
109	The use of diuretics	The use of inotropic agents	Without either tachycardia (100 bpm <) or bradycardia(<50 bpm)	The abnormal value of lvds (34 mm <) at admission	0.04356
110	The abnormal value of %FS (<30%) at admission	The use of inotropic agents	In NYHA class III or ivat admission		0.04356
111	The abnormal value of %FS (<30%) at admission	The use of inotropic agents	The abnormal value of lvds (34 mm <) at admission	In NYHA class III or ivat admission	0.04356
112	The abnormal value of %FS (<30%) at admission	The use of inotropic agents	The abnormal value of BNP (18.4 pg/ml <) at discharge	In NYHA class III or ivat admission	0.04356
113	The abnormal value of %FS (<30%) at admission	The use of inotropic agents	The use of diuretics	In NYHA class III or ivat admission	0.04356
114	The use of inotropic agents	Without either tachycardia (100 bpm <) or bradycardia(<50 bpm)	The abnormal value of lvds (34 mm <) at discharge		0.04356
115	The use of inotropic agents	Without either tachycardia (100 bpm <) or bradycardia(<50 bpm)	The abnormal value of bnp (18.4 pg/ml <) at discharge	The abnormal value of lvds (34 mm <) at discharge	0.04356
116	The use of diuretics	The use of inotropic agents	Without either tachycardia (100 bpm <) or bradycardia(<50 bpm)	The abnormal value of lvds (34 mm <) at discharge	0.04356
117	The use of inotropic agents	The abnormal value of lvds (34 mm <) at discharge	In nyha class iii or ivat admission	The abnormal value of lvds (34 mm <) at discharge	0.04356
118	The use of inotropic agents	The abnormal value of %fs (<30%) at discharge	Without either tachycardia (100 bpm <) or bradycardia(<50 bpm)		0.04356
119	The use of inotropic agents	The abnormal value of %fs (<30%) at discharge	Without either tachycardia (100 bpm <) or bradycardia(<50 bpm)	The abnormal value of bnp (18.4 pg/ml <) at discharge	0.04356
120	The use of diuretics	The use of inotropic agents	The abnormal value of %fs (<30%) at discharge	Without either tachycardia (100 bpm <) or bradycardia(<50 bpm)	0.04356
121	The use of inotropic agents	The abnormal value of %fs (<30%) at discharge	In nyha class iii or ivat admission	The abnormal value of lvds (34 mm <) at admission	0.04356
122	The abnormal value of %FS (<30%) at admission	The use of inotropic agents	The abnormal value of %FS (<30%) at discharge	In NYHA class III or ivat admission	0.04356
123	The use of inotropic agents	The abnormal value of %fs (<30%) at discharge	In nyha class iii or ivat admission	The abnormal value of lvds (34 mm <) at discharge	0.04356
124	The use of inotropic agents	The abnormal value of lvdd (52 mm <) at discharge	In nyha class iii or ivat admission		0.04356
125	The use of inotropic agents	The abnormal value of lvdd (52 mm <) at discharge	The abnormal value of bnp (18.4 pg/ml <) at discharge	In nyha class iii or ivat admission	0.04356
126	The use of diuretics	The use of inotropic agents	The abnormal value of lvdd (52 mm <) at discharge	In nyha class iii or ivat admission	0.04356
127	The abnormal value of %FS (<30%) at admission	The use of inotropic agents	The abnormal value of lvdd (52 mm <) at discharge		0.04356
128	The abnormal value of %FS (<30%) at admission	The use of inotropic agents	The abnormal value of lvdd (52 mm <) at discharge	The abnormal value of lvds (34 mm <) at admission	0.04356
129	The abnormal value of %FS (<30%) at admission	The use of inotropic agents	The abnormal value of lvdd (52 mm <) at discharge	The abnormal value of BNP (18.4 pg/ml <) at discharge	0.04356
130	The abnormal value of %FS (<30%) at admission	The use of inotropic agents	The abnormal value of lvdd (52 mm <) at discharge	The use of diuretics	0.04356
131	The abnormal value of %FS (<30%) at admission	The use of inotropic agents	The abnormal value of lvdd (52 mm <) at discharge	The abnormal value of lvds (34 mm <) at discharge	0.04356
132	The use of inotropic agents	The abnormal value of %fs (<30%) at discharge	The abnormal value of lvdd (52 mm <) at discharge		0.04356
133	The use of inotropic agents	The abnormal value of %fs (<30%) at discharge	The abnormal value of lvdd (52 mm <) at discharge	The abnormal value of lvds (34 mm <) at discharge	0.04356
134	The abnormal value of %FS (<30%) at admission	The use of inotropic agents	The abnormal value of %FS (<30%) at discharge	The abnormal value of lvdd (52 mm <) at discharge	0.04356
135	The use of inotropic agents	The abnormal value of %fs (<30%) at discharge	The abnormal value of lvdd (52 mm <) at discharge	The abnormal value of lvds (34 mm <) at admission	0.04356
136	The use of inotropic agents	The abnormal value of %fs (<30%) at discharge	The abnormal value of lvdd (52 mm <) at discharge	The abnormal value of bnp (18.4 pg/ml <) at discharge	0.04356
137	The use of diuretics	The use of inotropic agents	The abnormal value of %fs (<30%) at discharge	The abnormal value of lvdd (52 mm <) at discharge	0.04356
138	The abnormal value of %FS (<30%) at admission	The use of inotropic agents	The abnormal value of lvdd (52 mm <) at discharge		0.04356
139	The abnormal value of %FS (<30%) at admission	The use of inotropic agents	The abnormal value of lvdd (52 mm <) at discharge	The abnormal value of lvds (34 mm <) at admission	0.04356
140	The abnormal value of %FS (<30%) at admission	The use of inotropic agents	The abnormal value of lvdd (52 mm <) at discharge	The abnormal value of lvds (34 mm <) at discharge	0.04356
141	The abnormal value of %FS (<30%) at admission	The use of inotropic agents	The abnormal value of lvdd (52 mm <) at discharge	The abnormal value of lvdd (52 mm <) at discharge	0.04356
142	The abnormal value of %FS (<30%) at admission	The use of inotropic agents	The abnormal value of lvdd (52 mm <) at discharge	The abnormal value of BNP (18.4 pg/ml <) at discharge	0.04356
143	The abnormal value of %FS (<30%) at admission	The use of inotropic agents	The abnormal value of lvdd (52 mm <) at discharge	The use of diuretics	0.04356
144	The use of inotropic agents	The abnormal value of %fs (<30%) at discharge	The abnormal value of lvdd (52 mm <) at discharge		0.04356
145	The abnormal value of %FS (<30%) at admission	The use of inotropic agents	The abnormal value of %FS (<30%) at discharge	The abnormal value of lvdd (52 mm <) at discharge	0.04356
146	The use of inotropic agents	The abnormal value of %fs (<30%) at discharge	The abnormal value of lvdd (52 mm <) at discharge	The abnormal value of lvds (34 mm <) at admission	0.04356
147	The use of inotropic agents	The abnormal value of %fs (<30%) at discharge	The abnormal value of lvdd (52 mm <) at discharge	The abnormal value of lvds (34 mm <) at discharge	0.04356
148	The use of inotropic agents	The abnormal value of %fs (<30%) at discharge	The abnormal value of lvdd (52 mm <) at discharge	The abnormal value of lvdd (52 mm <) at discharge	0.04356
149	The use of inotropic agents	The abnormal value of %fs (<30%) at discharge	The abnormal value of lvdd (52 mm <) at discharge	The abnormal value of bnp (18.4 pg/ml <) at discharge	0.04356
150	The use of diuretics	The use of inotropic agents	The abnormal value of %fs (<30%) at discharge	The abnormal value of LVDd (52 mm <) at discharge	0.04356
151	The use of diuretics	Without either tachycardia (100 bpm <) or bradycardia(<50 bpm)	Living with family members in the same household		0.04969

**Table 4:** Summary of the results of LAMP procedure.

Category	The combination of clinical parameters				Number of the combination of clinical parameters
1	The use of inotropic agents				145
2	The use of diuretics	Without either tachycardia (100 bpm <) or bradycardia(<50 bpm)	In NYHA class III or IVat admission		1
	The use of diuretics	Without either tachycardia (100 bpm <) or bradycardia(<50 bpm)	In NYHA class III or IVat admission	The abnormal value of BNP (18.4 pg/ml <) at discharge	1
	The use of diuretics	Without either tachycardia (100 bpm <) or bradycardia(<50 bpm)	In NYHA class III or IVat admission	Living with family members in the same household	1
	The use of diuretics	Without either tachycardia (100 bpm <) or bradycardia(<50 bpm)			1
	The use of diuretics	Without either tachycardia (100 bpm <) or bradycardia(<50 bpm)	The abnormal value of BNP (18.4 pg/ml <) at discharge		1
	The use of diuretics	Without either tachycardia (100 bpm <) or bradycardia(<50 bpm)	Living with family members in the same household		1

**Fig. 1:**
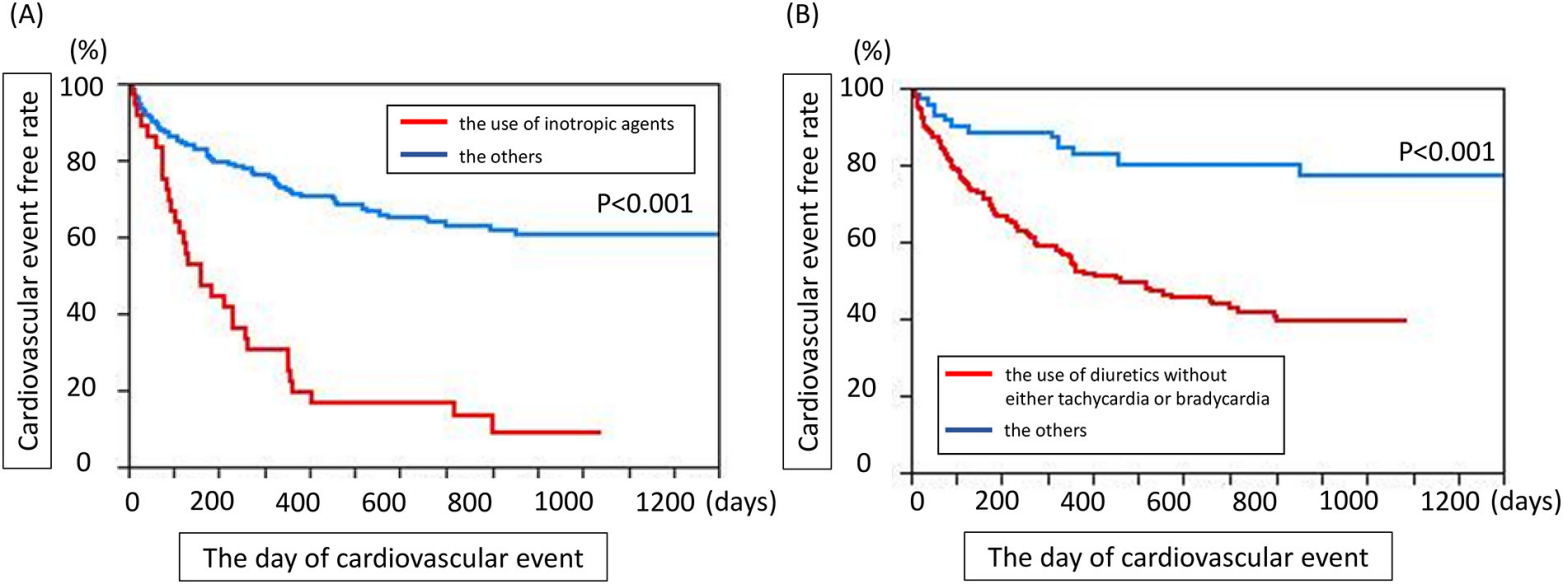
Kaplan-Meier curves for the cardiovascular events using the use of inotropic agents (A) and the use of diuretics without either bradycardia or tachycardia (B) in the HF patients.

Finally, we conducted a Kaplan–Meier analysis of these two clinical factors to determine whether they could accurately predict the occurrence of cardiovascular events in this patient population. Notably, both the use of inotropic agents and the use of diuretics without either tachycardia or bradycardia were strong and significant predictors of the occurrence of cardiovascular events among patients with HF (Fig. 1).

**Fig. 2:**
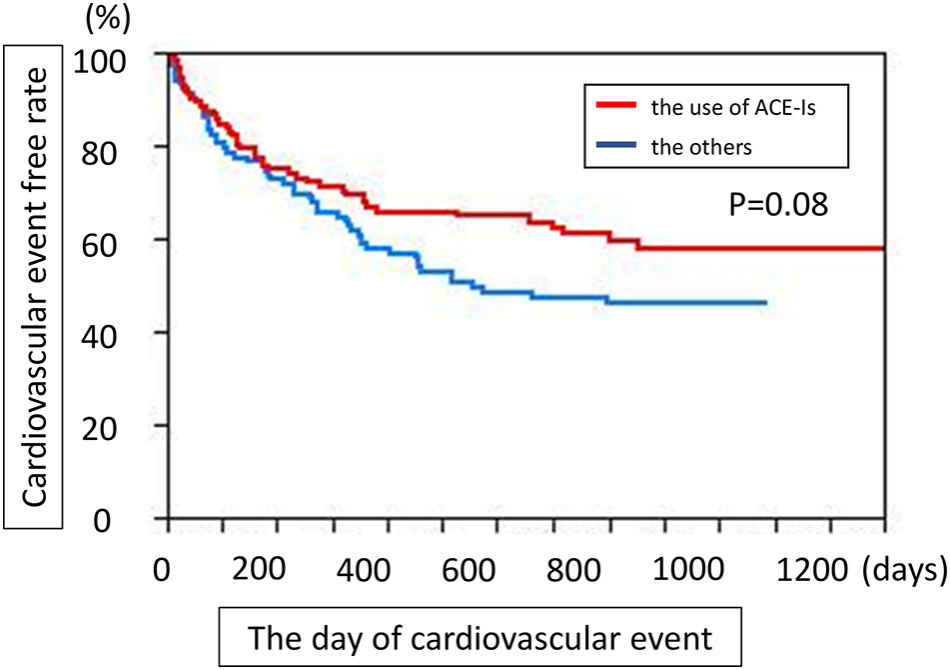
Kaplan-Meier curves for the cardiovascular events with and without the use of angiotensin converting enzymes (ACE-Is), the conventional and effective treatment of HF in the HF patients.

We further tested whether the approved treatment of HF such as angiotensin inhibitors (ACE-Is) is also found to be effective in the present cohort of the HF patients, and we found that ACE-Is seem to be effective in the prevention of cardiovascular events despite statistically insignificant levels of *p* = 0.08 (Fig. 2), indicating that the conventional and approved treatment strategies of HF patients seem to be effective in the present cohort. We further suggested that the use of pimobendan or the use of diuretics without either bradycardia or tachycardia more potently affects the severity of HF than ACE-Is, and may blunt the cardioprotective effects of ACE-Is.

## 4 Discussion

The effects of the present investigation are twofold. First, this study provides new pathophysiological evidence of the potential risk factors indicative of more severe HF; second, this research proposes a novel big data analysis strategy based on the new data-mining method, LAMP.

### 4.1 Ultimate Clinical Factors Affecting the Occurrence of Cardiovascular Outcomes

The present study has shown that either the use of inotropic agents or the use of diuretics without either bradycardia or tachycardia is a strong predictor of cardiovascular outcomes in patients with HF. Regarding the former factor, pimobendan was exclusively used in the present study because we considered digitalis to be an independent drug class rather than an inotropic agent. Indeed, a previous study found that although digoxin did not reduce the overall mortality, it reduced the rate of hospitalisation both overall and for worsening HF [16]. In the present study, the use of digitalis was not found to significantly reduce the incidence of cardiovascular events. By contrast, pimobendan was previously reported to improve the exercise capacity in patients with HF, although it was also associated with a 1.8-fold higher hazard of death [12]. Although pimobendan is often used for weaning from intravenous inotropic agents (e.g. PDE III inhibitors) [17], the present study suggests that this drug should not be administered to patients with HF. Furthermore, patients with HF who are already treated with pimobendan should be monitored carefully, given the high probability of the occurrence of the cardiovascular events.

As noted above, the use of diuretics also increased the risk of cardiovascular events among patients without either tachycardia or bradycardia. Consistent with our findings, a previous report described the difficulty of using diuretics to improve cardiovascular outcomes [18], and another study reported that vasodilators were superior to diuretics in terms of improved oxygen saturation and pulmonary ventilation [19]. In the present study, furosemide was the most frequently administered diuretic. However, furosemide may have the following detrimental effects: [1] exacerbation of renal dysfunction, [2] hyponatraemia and [3] activation of the renin–angiotensin and sympathetic nerve systems, which may worsen the clinical outcomes [20, 21]. These findings indicate that although diuretics may reduce symptoms, they do not improve cardiovascular outcomes [22].

Intriguingly, the second term specified diuretics ‘without either bradycardia or tachycardia’ as predictive of the occurrence of cardiovascular outcomes, leading us to wonder how the heart rate is involved; we were unable to determine an exact answer for this issue. Possibly, treatment with diuretics activates the sympathetic nervous system and, consequently, heart rate. Accordingly, the condition of diuretics without tachycardia may encompass patients in whom the sympathetic nerve system is exhausted even in the presence of diuretics (i.e. patients with more severe HF). Regardless of the underlying mechanism, we should focus on the present use of inotropic agents or the use of diuretics without either bradycardia or tachycardia as the strongest predictors of an increased risk of cardiovascular events in patients with HF.

### 4.2 Novel Mathematical Evaluation Protocol and Data-Centric Medicine

The present study has proposed the expediency of big data mining based on the LAMP [13] with the intent to identify unexpected single or combinational factors predictive of cardiovascular events. Briefly, data-mining methods are used to examine all possible combinations of all clinical parameters that might affect cardiovascular outcomes [23, 24]. This approach allowed us to employ and test both single and combinations of clinical parameters that might not appear to be directly linked to cardiovascular events. By contrast, a multivariate analysis evaluates the effects of each parameter on the clinical outcomes but cannot determine the effects of combinational factors. As noted above, LAMP minimises false negatives by calibrating the Bonferroni factor, maintains statistical power under multiple comparisons and provides the significant *p* values for each factor against the outcomes. Still, the factors identified using LAMP should be confirmed using ordinary statistical methods. In this study, we observed significantly different ratios of patients with and without cardiovascular events after dichotomising the patients according to each single or combinational factor (Fig. 1). Finally, these data-mining methods can be used in medical fields wherein cause–effect relationships are difficult to identify [25]. As for the required number of the data to be collected, there is no upper or lower limitation, however, when the data number is small, we cannot obtain the large number of the combination of the factors to explain the objective function.

### 4.3 Limitations of the Present Study

This study had a couple of noteworthy limitations. First, the study included a relatively small sample of patients. However, we achieved high levels of significance when we applied the use of inotropic agents or the use of diuretics without either bradycardia or tachycardia to determine the presence or absence of cardiovascular events, which suggests that the results in the present study are reliable. Additionally, our results were based on data from three Japanese hospitals that specialised in the treatment of HF. The results of the multicentre clinical trials are superior to those of the single center trails because the results of the multicetre clinical trials are more comprehensive. Interestingly, these three hospitals are Hokkaido University located in the north of Japan, National Cerebral and Cardiovascular Center at the center of Japan and Kyushu University at the southern part, which may guarantee the applicability of the present finding throughout Japan. One may argue that the present results may not be valid in other countries; however, as long as the pathophysiology and treatment strategy of HF are common worldwide, the present results should be valid to provide the future occurrence of cardiovascular events in other countries.

Second, we enrolled the moderate severity of the patients with HF in the present study, and the present results may not be applicable for very severe HF patients.

Third, it would be possible that the medications are given to sicker patients, and that the use of such medications may naturally predict the occurrence of the cardiovascular events. However, among measured many clinical parameters such as the BNP levels or used many drugs in HF patients, we found the use of pimobendan or the use of diuretics under the certain circumstance of heart rate only predicts cardiovascular events. What the present study suggest is that the patients treated with pimobendan or diuretics are very easily re-hospitalized due to the worsening of HF. Indeed, since pimobendan provided a 1.8-fold higher hazard of death in HF patients, we need to be careful to treat the HF patients with pimobendan. Although we cannot deny the possibility that pimobendan is used the severe HF patients, we are cautioned that we try not to use pimobendan for the HF patients.

Fourth, the use of beta-blockers or ACE-Is was not included among the strongest clinical parameters in the present study, although ACE-Is have some impacts on the prevention of cardiovascular events (Fig. 2). Although this finding might be expected to reduce the accuracy of the present study, both drugs are considered standard therapies for HF and are administered to many patients. Therefore, they no longer have a significant effect on the clinical outcomes. The other possibility is that the use of pimobendan or diuretics may confound the cardioprotective HF drugs such as ACE-Is in the cohort study, not in the randomised studies.

Taken together, these lines of evidence and consideration suggest that either the use of inotropic agents or the use of diuretics without either bradycardia or tachycardia culminated from the examination of all combination of the important clinical parameters is the strongest in predicting cardiovascular events in the HF patients in the contemporary era.

## 5 Conclusion

In conclusion, this analysis, which was based on the novel big data-mining technique, LAMP, identified the use of inotropic agents or the use of diuretics without either bradycardia or tachycardia as the most deleterious clinical parameters affecting patients receiving standard therapies for HF.

## References

[1] Braunwald E., Bristow M.R. (2000). Congestive heart failure: Fifty years of progress. Circulation.

[2] Ambrosy A.P., Fonarow G.C., Butler J. (2014). The global health and economic burden of hospitalizations for heart failure: Lessons learned from hospitalized heart failure registries. J Am Coll Cardiol.

[3] Thom T., Haase N., Rosamond W. (2006). Heart disease and stroke statistics—2006 update: A report from the American Heart Association Statistics Committee and Stroke Statistics Subcommittee. Circulation.

[4] Jessup M., Brozena S. (2003). Heart failure. N Engl J Med.

[5] Levy D., Kenchaiah S., Larson M.G. (2002). Long-term trends in the incidence of and survival with heart failure. N Engl J Med.

[6] McMurray J.J., Adamopoulos S., Anker S.D. (2012). ESC Guidelines for the diagnosis and treatment of acute and chronic heart failure 2012: The Task Force for the Diagnosis and Treatment of Acute and Chronic Heart Failure 2012 of the European Society of Cardiology. Developed in collaboration with the Heart Failure Association (HFA) of the ESC. Eur Heart J.

[7] Fukuda H., Suwa H., Nakano A. (2016). Non-linear equation using plasma brain natriuretic peptide levels to predict cardiovascular outcomes in patients with heart failure. Sci Rep.

[8] Kalogeropoulos A., Georgiopoulou V., Psaty B.M. (2010). Inflammatory markers and incident heart failure risk in older adults: The health ABC (health, aging, and body composition) study. J Am Coll Cardiol.

[9] Jackson C.E., Solomon S.D., Gerstein H.C. (2009). Albuminuria in chronic heart failure: Prevalence and prognostic importance. Lancet.

[10] Garg R., Yusuf S., Bussmann W.D. (1995). Overview of randomized trials of angiotensin-converting enzyme inhibitors on mortality and morbidity in patients with heart failure. JAMA.

[11] Felker G.M., Lee K.L., Bull D.A. (2011). Diuretic strategies in patients with acute decompensated heart failure. N. Engl. J. Med..

[12] Lubsen J., Just H., Hjalmarsson A.C. (1996). Effect of pimobendan on exercise capacity in patients with heart failure: Main results from the pimobendan in Congestive Heart Failure (PICO) trial. Heart (British Cardiac Society).

[13] Terada A., Okada-Hatakeyama M., Tsuda K., Sese J. (2013). Statistical significance of combinatorial regulations. Proc Natl Acad Sci U S A.

[14] Yoshida A., Asakura M., Asanuma H. (2013). Derivation of a mathematical expression for predicting the time to cardiac events in patients with heart failure: A retrospective clinical study. Hypertens. Res..

[15] Sakamoto M., Fukuda H., Kim J. (2018). The impact of creating mathematical formula to predict cardiovascular events in patients with heart failure. Sci Rep.

[16] The effect of digoxin on mortality and morbidity in patients with heart failure. The (1997). N. Engl. J. Med..

[17] Endoh M., Hori M. (2006). Acute heart failure: Inotropic agents and their clinical uses. Expert Opin Pharmacother.

[18] Shah M.R., Stevenson L.W. (2004). Searching for evidence: Refractory questions in advanced heart failure. J Card Fail.

[19] Cotter G., Metzkor E., Kaluski E. (1998). Randomised trial of high-dose isosorbide dinitrate plus low-dose furosemide versus high-dose furosemide plus low-dose isosorbide dinitrate in severe pulmonary oedema. Lancet.

[20] Bayliss J., Norell M., Canepa-Anson R., Sutton G., Poole-Wilson P. (1987). Untreated heart failure: Clinical and neuroendocrine effects of introducing diuretics. Br Heart J.

[21] Petersen J.S., DiBona G.F. (1994). Reflex control of renal sympathetic nerve activity during furosemide diuresis in rats. Am J Physiol.

[22] Yancy C.W., Jessup M., Bozkurt B. (2013). 2013 ACCF/AHA guideline for the management of heart failure: A report of the American College of Cardiology Foundation/American Heart Association Task Force on Practice Guidelines. J Am Coll Cardiol.

[23] Podgorelec V., Kokol P., Stiglic B., Rozman I. (2002). Decision trees: An overview and their use in medicine. J Med Syst.

[24] Kim J., Washio T., Yamagishi M. (2004). A novel data mining approach to the identification of effective drugs or combinations for targeted endpoints—application to chronic heart failure as a new form of evidence-based medicine. Cardiovascular Drugs Ther..

[25] Hey T., Tansley S., Tolle K.M. (2009). The fourth paradigm: Data-intensive scientific discovery.

